# A pilot study of alterations in oxidized angiotensinogen and antioxidants in pre-eclamptic pregnancy

**DOI:** 10.1038/s41598-020-58930-7

**Published:** 2020-02-06

**Authors:** Lina A. Dahabiyeh, David Tooth, Lesia O. Kurlak, Hiten D. Mistry, Fiona Broughton Pipkin, David A. Barrett

**Affiliations:** 1 Department of Pharmaceutical Sciences, School of Pharmacy, The University of Jordan, Amman, Jordan; 2 Centre for Analytical Bioscience, Division of Advanced Materials and Healthcare Technologies, School of Pharmacy, University of Nottingham, Nottingham, UK; 3 BBSRC/EPSRC Synthetic Biology Research Centre, School of Life Sciences, University of Nottingham, Nottingham, UK; 4 Division of Child Health, Obstetrics and Gynaecology, School of Medicine; University of Nottingham, Nottingham, UK

**Keywords:** Proteomics, Biochemistry, Enzymes, Oxidoreductases, Hypertension, Pre-eclampsia, Diagnostic markers, Medical research, Preclinical research

## Abstract

The oxidation status of angiotensinogen (AGT) may have a critical role in pre-eclampsia. We used a validated, quantitative, mass spectrometry-based method to measure the oxidized and total AGT levels in plasma of pre-eclamptic women (n = 17), normotensive-matched controls (n = 17), and healthy non-pregnant women (n = 10). Measurements of plasma glutathione peroxidase (GPx) activity and serum selenium concentrations were performed as markers of circulating antioxidant capacity. Higher proportions of oxidized AGT in plasma from pre-eclamptic women compared to matched normotensive pregnant controls (P = 0.006), whilst maintaining a similar total plasma AGT concentration were found. In the pre-eclamptic group, blood pressure were correlated with the proportion of oxidized AGT; no such correlation was seen in the normotensive pregnant women. Plasma GPx was inversely correlated with oxidized AGT, and there was an inverse association between serum selenium concentration and the proportion of oxidized AGT. This is the first time that oxidized AGT in human plasma has been linked directly to antioxidant status, providing a mechanism for the enhanced oxidative stress in pre-eclampsia. We now provide pathophysiological evidence that the conversion of the reduced form of AGT to its more active oxidized form is associated with inadequate antioxidant status and could indeed contribute to the hypertension of pre-eclampsia.

## Introduction

Pre-eclampsia is conventionally defined as new-onset gestational hypertension and significant proteinuria^1^, accompanied by widespread endothelial damage leading to maternal organ dysfunction such as renal insufficiency, neural or haematological complications. It is one of the leading causes of maternal and fetal morbidity and mortality worldwide, not only in low- and middle-income countries^2^. but also in such highly-developed countries as the Netherlands, where it is the leading cause of maternal death^3^.

The cause of pre-eclampsia is multi-faceted. Increasingly, it is realised that underlying maternal factors, such as genetic make-up^4^ and diet, particularly antioxidant micronutrients^5^, can predispose to the condition. There is considerable interest in the effect of reactive oxygen species (ROS), which include free radicals such as superoxide anions, nitric oxide and hydroxyl radicals, in disrupting the biological function of endogenous proteins^6^. When these free radicals are not suitably dampened by antioxidants, this can result in oxidative stress^7^. Selenium is a trace element, essential in the synthesis of a number of antioxidant enzymes, including the selenoprotein, glutathione peroxidases (GPxs). There are six antioxidant GPxs, which are central to the reduction of hydrogen peroxide (H_2_O_2_) and lipid peroxides to harmless products (water and alcohols), thus lowering free radical generation. Through their antioxidant activity, the GPxs help maintain membrane integrity, protect prostacylin production, and limit oxidative damage to lipids, lipoproteins and DNA^8^. We and others have reported that pregnant women in the UK have low selenium status and GPx activities and those with pre-eclampsia have still lower values^9,10^.

Placental oxidative stress, initiated as a consequence of poor implantation and impaired remodelling of the uterine spiral arteries^11,12^ and extending to the general circulation, is in turn associated with inappropriate up-regulation of antiangiogenic factors, endothelial damage is believed to be central to the pathogenesis of pre-eclampsia^13^.

The renin-angiotensin system (RAS) is one of the first endocrine systems to “recognize” pregnancy^14,15^. Outside pregnancy, it is the concentration of renin, which is rate-limiting in the release of angiotensin I (Ang I) from angiotensinogen (AGT), but in pregnancy, the concentration of AGT becomes rate-limiting^16^. A recent large study, using an integrated systems biology approach, identified AGT as having a central role in trophoblastic pathology in pre-term pre-eclampsia^17^. Dysregulation in the RAS has been closely linked to the formation of excessive amounts of free radicals, resultant oxidative stress and thus breakdown of the mitochondrial reduction–oxidation balance^18^. The oxidative state in pre-eclampsia is proposed to enhance the conversion of AGT in the maternal circulation to the more active oxidized form by the formation of a disulphide-bridge between Cys 18 and Cys 138^19^. The oxidized form of AGT, compared to the reduced form, interacts with renin with four-fold higher binding affinity resulting in increased generation of Ang I, and hence angiotensin II (Ang II) in the presence of similar absolute AGT concentrations^19^. Two previous studies, one using Western blotting and the other ELISA, both reported a significant decrease in the level of the reduced form of AGT, reflecting a higher level of the oxidized form, in the plasma of pre-eclamptic women compared to controls^20,21^. However, antibody-based assays suffer from poor selectivity to discriminate between different protein isoforms^22^. Furthermore, our group have reported that total plasma AGT concentrations are not different between pre-eclampsia and normotensive controls^23^. We have, therefore, recently developed a more selective, quantitative mass spectrometry (MS)-based method for measuring the oxidized and reduced forms of AGT and the total concentration of AGT in human plasma^24^.

The samples used to investigate possible differences in the proportion of oxidized AGT in established pre-eclampsia^21^, were part of a detailed study of selenium and related antioxidant micronutrients^10^. In order to determine whether a study primarily designed to examine the influence of such mechanisms on the proportion of oxidized AGT might be justified, we measured the proportion of oxidised AGT, using our new methodology^24^ in these samples.

We now report data obtained using this method to measure the oxidized and the total levels of AGT in the plasma of these pre-eclamptic women, matched controls and normal non-pregnant women. The aims were first: using our accurate quantification of the oxidation level of AGT to confirm the observation that the proportion of oxidized AGT is higher in pre-eclamptic women than in normotensive pregnant women and second: to provide pilot clinical data testing the hypothesis that the inadequate endogenous antioxidants reported in pre-eclampsia, resulting in increased oxidative stress, would be directly associated with the proportion of oxidized AGT, and that, in turn, the proportion of oxidized AGT would be associated with the maternal blood pressure.

## Results

Table 1 describes the demographic characteristics of the 34 pre-eclampsia cases and controls and the 10 non-pregnant women who participated in this study. No significant differences were detected between pre-eclampsia and matched control groups with regards to blood pressure at booking, delivery method, maternal age, parity, or body mass index (BMI).

**Table 1:** Maternal demographic and clinical characteristics of pre-eclampsia cases and their matched controls.

Maternal Characteristics	Non-pregnant (*n* = 10)	Normotensive pregnant (*n* = 17)	Pre-eclampsia (*n* = 17)
Age (years)	33.4 ± 11.3	29.3 ± 6.8	32.5 ± 6.0
BMI at booking (kg/m^2^)	22.1 ± 1.8	25.6 ± 4.9	26.7 ± 5.8
Systolic blood pressure at booking (mm Hg)	—	113.7 ± 11.4	122.6 ± 8.9
Diastolic blood pressure at booking (mm Hg)	—	68.7 ± 10.6	73.6 ± 11.7
Systolic blood pressure outside labour (mm Hg)	—	115.9 ± 4.4	158 ± 11.3*
Diastolic blood pressure outside labour (mm Hg)	—	75.5 ± 2.8	97.9 ± 5.3*
Gestation age at delivery (weeks)	—	39.7 ± 1.2	36.4 ± 4.3*
Caesarean section, n (%)	—	3 (17.6%)	6 (35.3%)
Birth weight (kg)	—	3.49 ± 0.43	2.78 ± 1.16*

Data are presented as mean ± SD except for caesarean sections (No. (percentage)).

^*^P < 0.05 between normotensive and pre-eclamptic pregnancies.

Both pregnancy groups conceived spontaneously and carried singleton pregnancies. The normotensive pregnancy controls all delivered healthy babies without any pregnancy complications and gave birth to infants weighing >2500 g, delivered 38 weeks or later. Women in the pre-eclampsia group all had moderate to severe disease, without HELLP, and had significantly higher blood pressure, lower gestational ages at delivery and lower infant birth weight than the control group (Table 1). All neonates from both pregnancy groups survived.

### Measurement of AGT concentration in the plasma of non-pregnant and normotensive and pre-eclamptic pregnant women

**Figure 1:**
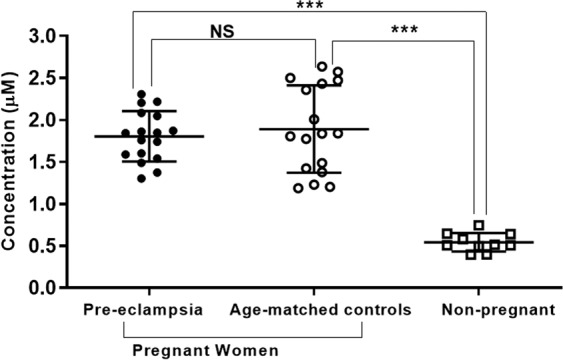
Differences in concentration of AGT in the plasma of pregnant women (pre-eclampsia and age-matched controls) and non-pregnant women. Data presented as mean ± SD of AGT concentration. NS: non-significant difference, ***P < 0.001.

Pre-eclamptic women and age-matched controls showed a 3.3 and a 3.5 fold, respectively increase in the level of circulating AGT compared to non-pregnant women (P < 0.001; P < 0.001 respectively). AGT concentrations in pregnant women were in the micro-molar range with a mean ± SD plasma concentration of 1.80 ± 0.4 and 1.89 ± 0.6 µM for pre-eclampsia and normotensive controls respectively, whereas normal non-pregnant women had a plasma AGT concentration of 0.544 ± 0.2 µM (Fig. 1). When calculated based on the AGT molecular weight (60 kDa), similar differences were observed, with the pre-eclamptic women (108.7 ± 17.5 µg/mL) and age-matched normotensive controls (113.5 ± 31.2 µg/mL) having increased mean circulating AGT concentration compared to non-pregnant women (32.7 ± 6.5 µg/mL; P < 0.001 between pregnant and non-pregnant samples).

The mean level of AGT in pregnancy was comparable in the pre-eclampsia group and their normotensive pregnant controls (P > 0.05; Fig. 1).

### Measurement of oxidized AGT in the plasma of normal and pregnant women (pre-eclampsia and controls)

**Figure 2:**
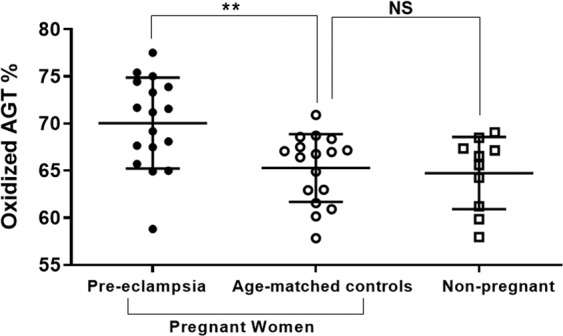
The proportion of AGT oxidation in the plasma of pre-eclamptic, controls and normal non-pregnant women. Data presented as mean ± SD of the percentage of oxidized AGT. NS: non-significant difference, **P = 0.006.

Women with pre-eclampsia showed a higher percentage of oxidized AGT in their plasma (70.0 ± 4.8%) when compared to normotensive pregnant controls (65 ± 3.6%) and normal non-pregnant women (64.7 ± 4.0%). Although the increase in the level of oxidized AGT in the plasma of pre-eclamptic women was relatively small it was still statistically significant (P = 0.006; Fig. 2). Pregnant controls and non-pregnant women showed very similar levels of oxidized AGT in their plasma and no significant difference was detected (P = 0.942; Fig. 2). Specific peptide LC-MS/MS data is presented in Supplementary Figs. S1 and S2.

### Clinical correlates with oxidized AGT

**Figure 3:**
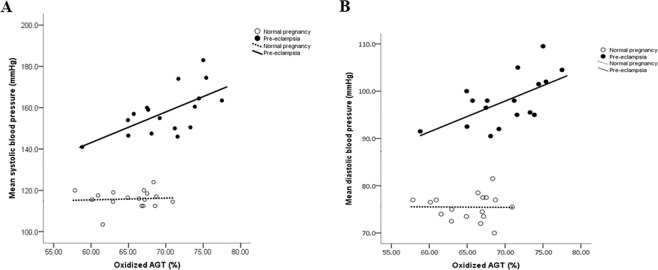
Scatter plot illustrating highly significant correlations between the proportion of oxidized AGT and both systolic (**A**: r = 0.676, P < 0.003) and diastolic (**B**: r = 0.552, P = 0.022) blood pressure in the pre-eclamptic, but not in the normotensive, pregnant women.

Given the increased capacity of renin to cleave oxidized AGT to release Ang I, we examined possible correlations between the systolic and diastolic blood pressures and the proportion of oxidized AGT in the pregnant women. In the pre-eclamptic group, both systolic and diastolic blood pressure were significantly correlated with the proportion of oxidized AGT (systolic: r = 0.676, P < 0.003; diastolic: r = 0.552, P = 0.022); Fig. 3A,B), whereas no such correlation was seen in the normotensive pregnant women (P > 0.8 for both).

**Figure 4:**
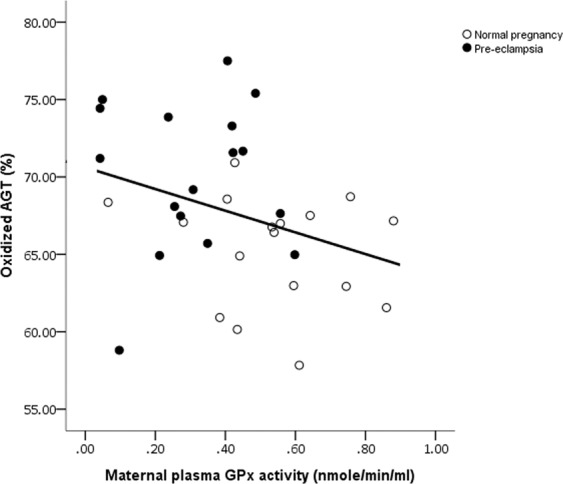
Scatter plot illustrating statistically significant inverse correlation between maternal plasma GPx activity and the proportion of oxidized AGT both over all women studied (r = −0.354, P = 0.018) and when only the two pregnant groups were studied (r = −0.349, P = 0.043; as shown).

Plasma activities of GPx were higher in non-pregnant (0.31 [0.15, 0.44]), compared to both normotensive pregnant women (0.79 [0.66, 0.90] nmol/min/ml; P < 0.0001) and pre-eclamptic women (0.54 [0.42, 0.70] nmol/min/ml; P < 0.0001). Moreover, normotensive control women had higher activities than pre-eclamptic women (P < 0.0001). Plasma GPx was inversely correlated with the proportion of oxidized AGT both overall (r = −0.354, P = 0.018; Fig. 4) and when only the two pregnancy groups were considered (r = −0.349, P = 0.043), but not when separated by normotensive control and those with pre-eclampsia (P > 0.05).

Serum selenium concentrations were: median [IQR]: 69.5 [61.2, 73.6], 62.9 [49.0, 68.2] and 40.4 [29.8, 48.6] µg/L in non-pregnant controls, normotensive pregnant women and pre-eclamptic women respectively (P < 0.001; Supplementary Fig. S3). The selenium concentration was significantly lower in pre-eclamptic women than in either non-pregnant controls (P < 0.0001) or normotensive pregnant women (P = 0.02). Selenium concentrations between non-pregnant controls and normotensive controls was not statistically-significant (P > 0.1). There was an inverse association overall, between serum selenium concentration and the proportion of oxidized AGT (r = −0.321, P = 0.036).

## Discussion

A review in 1983 drew attention to the variety of physiological and pathological states in humans and animals in which positive correlations between the blood pressure and AGT concentrations were observed^25^. Our results, using LC-MS/MS analysis, confirmed a significantly higher plasma concentration of AGT during pregnancy (pre-eclampsia and normotensive pregnancy) compared to non-pregnant women. We have also identified, for the first time, strongly positive correlations between the proportion of oxidized AGT and systolic and diastolic blood pressure in pre-eclamptic, but not normotensive, human pregnancy. Furthermore, this new method has validated the previous Western blot data^19^ reporting increased plasma oxidized AGT in women with pre-eclampsia, compared to matched-normotensive pregnant controls.

It is possible that the correlation between blood pressure and the proportion of oxidized AGT in hypertensive pregnancies is due simply to the four-fold greater Ang I-generating capacity of oxidized AGT^19^ and consequently higher concentrations of Ang II. The measurement of Ang II in plasma is fraught with difficulty, especially in pregnancy, because of its short half-life (<50 sec) in the circulation^26^. The circulating concentration of angiotensinases rises in pregnancy, so that blood samples should be taken into an enhanced cocktail of chilled angiotensinase inhibitors and the angiotensins extracted from plasma as rapidly as possible^27^. These precautions are rarely taken in clinical studies. Ang II was not measured in this study, but an earlier study, using plasma handling techniques described above, did identify higher concentrations of circulating Ang II in hypertensive primigravidae, which correlated with diastolic blood pressure^28^. It has been known for more than 40 years^29^, that blockade of the RAS in pregnancy does indeed result in a fall in maternal blood pressure, but the adverse effects on the fetus preclude the use of such blockade in pre-eclampsia.

There is considerable interest in the role of the balance between pro-angiogenic (e.g. vascular endothelial growth factor (VEGF) and placental growth factor (PlGF)) and anti-angiogenic (e.g. soluble fms-like tyrosine kinase-1 (sFlt-1)) factors in pre-eclampsia^30^; Ang II appears to act upstream of these factors. Blockade of the Ang II Type 1 receptors (AT1Rs) in a first trimester human placental cell line (HTR-8/SVneo), incubated at low oxygen concentrations, significantly reduced the induced rise in both intracellular and secreted VEGF-A^31^. Conversely, while the secretion of sFlt-1 from placental explants from near-term pregnant rats, subjected to reduced uterine perfusion pressure was increased; the rise was abolished when animals had simultaneously been given an AT1R blocker, further suggesting the association of Ang II acting upstream. The induced rise in mean arterial blood pressure was also significantly attenuated^32^.

**Figure 5:**
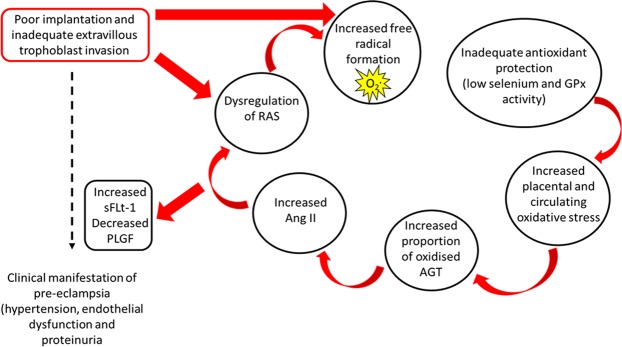
Proposed mechanism linking dysregulation of the renin-angiotensin system (RAS), with inadequate selenium and antioxidant protection, leading to pre-eclampsia. Poor implantation, inadequate evtravillous trophoblast invasion and resultant poor placental perfusion could lead to dysregulation in the RAS. The RAS has been closely linked to the formation of excessive amounts of free radicals and thus breakdown of the mitochondrial reduction–oxidation balance. This extra free radical production, with the known reduced selenium and reduced antioxidant glutathione peroxidase (GPx) activity in pre-eclampsia will result in increased placental and circulating oxidative stress. This oxidative state is proposed to enhance the conversion of AGT in the maternal circulation to the more active oxidized form. The oxidized form of AGT, compared to the reduced form, interacts with renin with four-fold higher binding affinity resulting in increased generation of angiotensin I, and hence the potent vasoconstrictor angiotensin II (Ang II) in the presence of similar absolute AGT concentrations. Finally, this further disrupts the RAS as well as alters downstream antiangiogenic makers through increased sFLt-1 and reduced placental growth factor (PLGR), culminating the in the clinical manifestation of pre-eclampsia.

In the study, plasma selenium and GPx concentrations were significantly decreased in pre-eclamptic women, and both were significantly inversely correlated with the proportion of oxidized AGT, providing a physiological basis for this increased oxidation. A summary of our proposed mechanism is presented in Fig. 5. The RAS is activated in every ovulatory menstrual cycle, and, should conception occur, the activation is maintained, making the RAS one of the earliest hormone systems to recognise pregnancy^14,15^. miRNAs, which target the placental RAS control trophoblast proliferation *in vitro*^33^ and their expression is modulated by the low oxygen tension prevailing at that time. The effects of oxidative stress in the peri-implantation period may themselves interfere with this balance. Interestingly, a recent, multi-faceted, integrated systems biological approach also identified AGT as being one of the central players in pre-term pre-eclampsia^17^.

A limitation of the current study was the availability of limited samples, however we were still able to demonstrate significant differences and confirmed our previous Western blot data^21^, which used lower numbers per group. We collected sequential samples from pre-eclamptic women. Six of these delivered before 37 weeks (preterm). We did consider subgroup analysis, but felt that there were insufficient pre-term deliveries to justify this. Visual inspection did however suggest a further increase in the proportion of oxidized angiotensinogen in pre-eclamptic women delivering prematurely, by comparison with those delivering at term. This will be further considered in our on-going study.

In conclusion, this is the first time that the proportion of oxidized AGT in human plasma has been linked directly to antioxidant status, thus providing a mechanism for the enhanced oxidation reported in pre-eclampsia. Antioxidant status is poor in women with pre-eclampsia. Dietary interventions to increase antioxidant intake could be an acceptable and relatively cheap means of lowering the oxidation of AGT, so reducing or removing this pathway towards hypertension.

## Material and Methods

### Power calculations

We calculated that a sample size of a minimum 10 in each group (healthy non-pregnant women, pre-eclamptic, age-matched normotensive pregnant women) would give us an 85% power of detecting a 20% difference in the proportion if oxidized AGT, using our previous western blot data^21^.

### Patient recruitment

The study population consisted of 3 groups of women: 10 healthy non-pregnant women of reproductive age, 17 pre-eclamptic, and 17 age-matched normotensive pregnant women. All pregnant blood samples were collected in the 3^rd^ trimester, before onset of labour. Pre-eclampsia was stringently defined^34^; women in the pre-eclampsia group had normal booking blood pressures at the first antenatal clinic visit (12–20 weeks), subsequently developed blood pressures of ≥140 mmHg systolic or ≥90 mmHg diastolic on two occasions (minimum 24 h apart) and (3) proteinuria ≥300 mg/L or 2+ on dipstick analysis. Age-matched control women had a normotensive blood pressure throughout their pregnancy and did not develop any other complication. Any women with pre-existing hypertension, diabetes or any other placenta-related complications were excluded.

### Plasma sample collection and processing

Peripheral blood samples from pre-eclamptic and age-matched normotensive pregnancies and healthy non-pregnant women were collected after obtaining fully informed written consent from each participant. Ethical approval for the sample collection and utilisation was given by the Hospital Ethics Committee of the Nottingham University Hospitals (OG 090301, LREC2 Q2090312). All methods were carried out in accordance with the Declaration of Helsinki.

Blood samples were collected into chilled tubes containing EDTA for AGT analysis and GPx activities and plain tubes for serum selenium concentrations. Samples were centrifuged after collection at 1000 × *g* for 15 min at 4 °C. The separated plasma and serum were then transferred to Eppendorf tubes in 0.5 mL aliquots, snap-frozen and stored at −80 °C until analysis.

### Sample preparation and analysis by liquid chromatography-tandem mass spectrometry (LC-MS/MS)

Plasma sample preparation and analysis by LC-MS/MS were performed as previously described^24^. Briefly, AGT was selectively extracted from plasma (50 µL) using 2-dimensional chromatography employing Concanavalin A lectin affinity and reversed-phase steps. Enriched AGT samples were deglycosylated using PNGase F followed by differential alkylation of the reduced and the oxidized form of AGT using ^13^C_0_,D_0_- and ^13^C_2_,D_2_-iodoacetamide respectively. Protein digestion was carried out using sequencing grade chymotrypsin. Peptides were analysed by a Shimadzu series 10AD VP LC system (Shimadzu, Columbia, MD) using a C18, 300 Å, 100 × 1 mm, 3 µm column (ACE, Reading, UK) and a mobile phase of water and acetonitrile both with 0.1% formic acid. Chymotrypsin-produced signature peptides were characterized using a 4000 QTRAP hybrid triple quadrupole/linear ion trap mass spectrometer (SCIEX, Foster City, CA, USA). Multiple reaction monitoring (MRM) transitions were monitored for the following AGT signature peptides: (1) AGT marker peptide (SVTQVPF), which was used to infer the plasma level of total AGT; (2) ^13^C_0_,D_0_-iodoacetamide alkylated Cys18 peptide (HLVIHDESTC^18^EQL), which corresponds to the reduced form of plasma AGT and (3) ^13^C_2_,D_2_*-* iodoacetamide alkylated Cys18 peptide (HLVIHDESTC^18^EQL), which corresponds to the oxidized form of plasma AGT.

### Measurement of the total and the oxidixed levels of plasma AGT

The concentrations of plasma AGT were determined for the three studied groups, after correcting for recovery using the internal standard method^24^.

To provide a quantitative comparison of the oxidation level of AGT in the plasma, the level of Cys18 peptide oxidation was calculated and compared between the three conditions (pre-eclampsia, controls and non-pregnancy). The level of Cys18 peptide oxidation was calculated as the percentage of the peak area of the oxidized Cys18 peptide (alkylated with ^13^C_2_,D_2_*-*ioodacetamide*)* over the sum of the reduced Cys18 peptide (alkylated with ^13^C_0_,D_0_*-*iodoacetamide*)* and oxidized Cys18 peptide as shown below:

$${\rm{Oxidized}}\,{\rm{AGT}} \% =\frac{{\rm{Oxidized}}\,{\rm{Cys}}\,{\rm{peptide}}\,}{{\rm{Oxidized}}\,{\rm{Cys}}\,{\rm{peptide}}\,+{\rm{Reduced}}\,{\rm{Cys}}\,{\rm{peptide}}\,}\times 100 \% $$

### Other biochemical measurements

Plasma GPx activity was measured as detailed previously^10^; the inter- and intra-assay variations were <5%. Serum selenium concentrations were determined by graphite furnace atomic absorption spectrophotometry, as previously described^10^. The intra- and inter-assay coefficients of variances were <5% for both with a lower detection limit of 0.3 μg/L. The measured serum selenium concentration of one patient in the pre-eclamptic group (115.8 μg/L) was more than 3xSD above the mean for all pregnant samples (50.2 + /−17.2 μg/L) and more than 5xSD above the mean for all other pre-eclamptic women (41.9 +/− 14.4 μg/L)) and was thus not included in statistical analysis.

### Statistical methods

All data are presented as the mean ± standard deviation (SD) or median (IQR) as appropriate. Phenotypic data for pre-eclamptic and control women were compared using a two-tailed independent *t*-test for continuous parameters, and Pearson Chi-square for comparative analysis of categorical variables. One way ANOVA (with post hoc Tukey HSD test if significant) was used to compare the plasma levels of the total and the oxidized AGT between the three studied groups. Kruskal Wallis ANOVA was used to analyse GPx activity across groups. Statistical analysis was performed with the Statistical Package for Social Sciences version 22.0 (SPPS Inc., Chicago, IL, USA) and GraphPad Prism 6 (GraphPad Software, Inc., La Jolla, CA 92037 USA). The Null Hypothesis was rejected at P < 0.05.

## Supplementary information

Supplementary informations.
