# Supplementary material for: A pilot study of alterations in oxidized angiotensinogen and antioxidants in pre-eclamptic pregnancy
Source: Sci Rep. 2020 Feb 6;10:1956. doi: 10.1038/s41598-020-58930-7 (PMC7004983; doi:10.1038/s41598-020-58930-7)
Supplement: Supplementary file 1 — Supplementary informations. [file 41598_2020_58930_MOESM1_ESM.pdf]

A pilot study of alterations in oxidized angiotensinogen and antioxidants in pre-eclamptic pregnancy

Lina A. Dahabiyeh[1,2], David Tooth[3], Lesia O. Kurlak[4], Hiten D. Mistry[4], Fiona Broughton Pipkin[4*] and David A. Barrett[2]

[1]Department of Pharmaceutical Sciences, School of Pharmacy, The University of Jordan, Jordan.

[2]Centre for Analytical Bioscience, Division of Advanced Materials and Healthcare Technologies, School of Pharmacy, University of Nottingham, UK; [3]BBSRC/EPSRC Synthetic Biology Research Centre, School of Life Sciences, University of Nottingham, UK; [4]Division of Child Health, Obstetrics and Gynaecology, School of Medicine; University of Nottingham, UK

Supplementary Figures

Figure S1

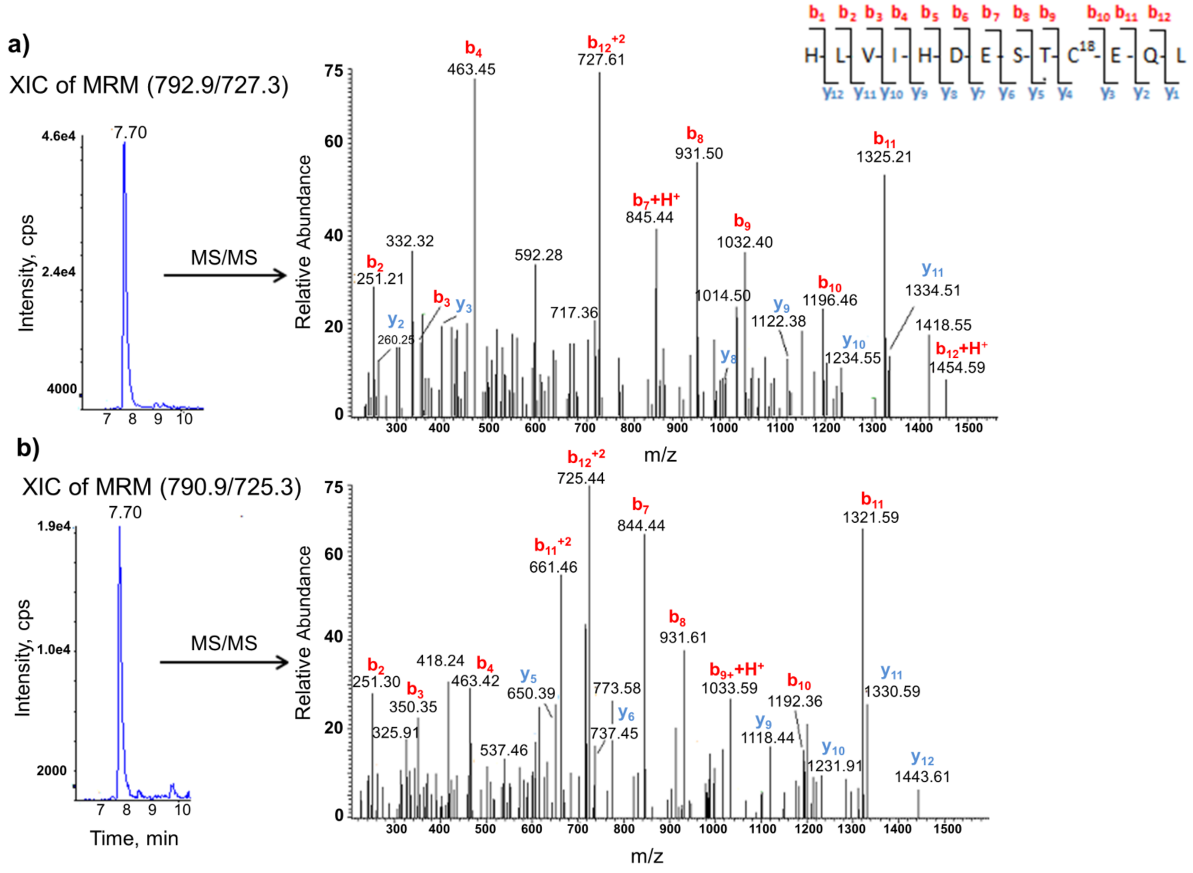

The extracted ion chromatogram (XIC) of multiple reaction monitoring (MRM) and the corresponding MS/MS spectra for the differentially alkylated Cys18 peptides detected in human plasma digest. a) Cys18 peptide alkylated with isotope labeled 13C2,D2-iodoacetamide representing the oxidised form of AGT in the plasma. b) Cys18 peptide alkylated with unlabeled 13C0,D0-iodoacetamide representing the reduced form of AGT in the plasma. Peptide identity and differential alkylation were confirmed by MS/MS spectra.

Figure S2

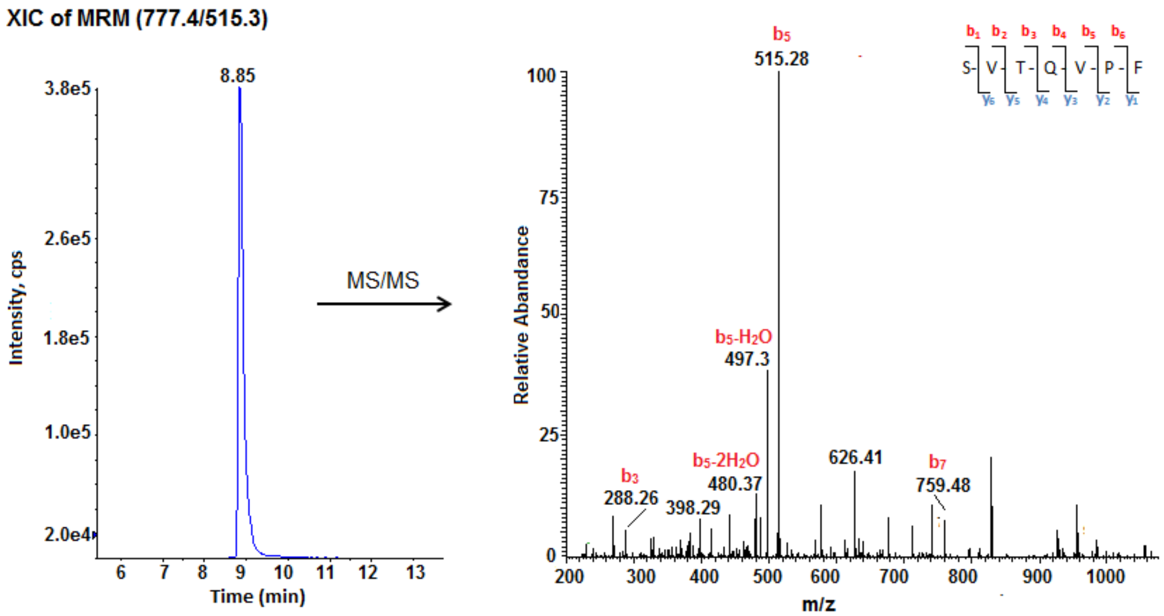

The XIC of MRM and the corresponding MS/MS spectrum for AGT marker peptide detected in the plasma digest of human plasma sample. Peptide identity was confirmed by MS/MS spectrum.

Figure S3

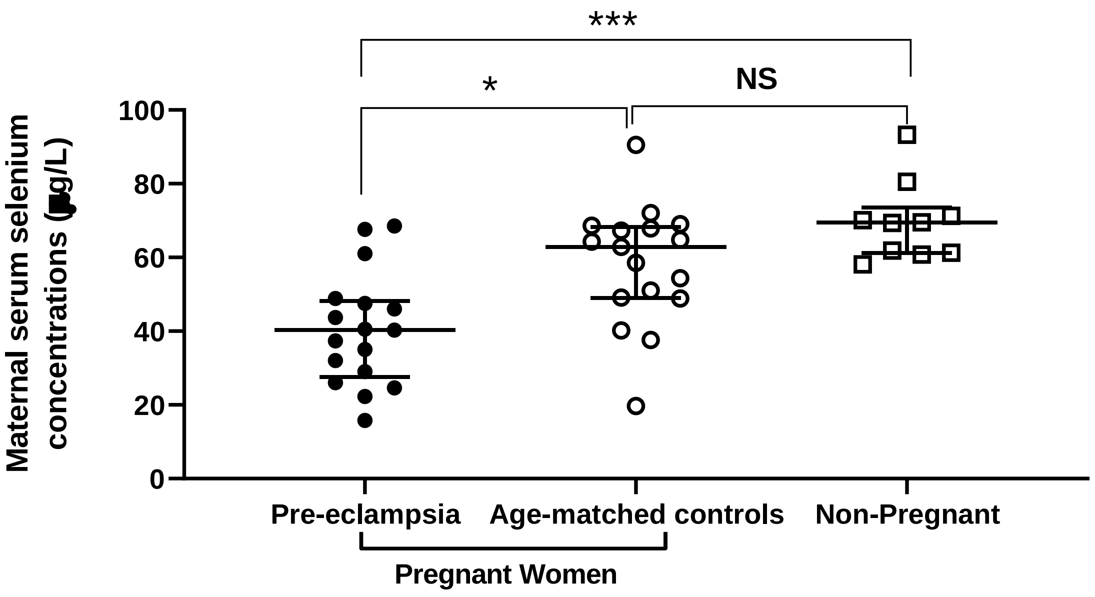

Maternal selenium concentrations from serum in pregnant (pre-eclampsia and age-matched controls) and non-pregnant women. Data presented as median [IQR] of selenium concentration (μg/L). NS: non-significant difference, *P<0.05; ***P<0.0001.
